# Resistance Mutations outside the Integrase Coding Region Have an Effect on Human Immunodeficiency Virus Replicative Fitness but Do Not Affect Its Susceptibility to Integrase Strand Transfer Inhibitors

**DOI:** 10.1371/journal.pone.0065631

**Published:** 2013-06-11

**Authors:** Jan Weber, Justine D. Rose, Ana C. Vazquez, Dane Winner, Nicolas Margot, Damian J. McColl, Michael D. Miller, Miguel E. Quiñones-Mateu

**Affiliations:** 1 Institute of Organic Chemistry and Biochemistry, Prague, Czech Republic; 2 Department of Biochemistry, Case Western Reserve University, Cleveland, Ohio, United States of America; 3 School of Biomedical Sciences, Kent State University, Kent, Ohio, United States of America; 4 University Hospital Translational Laboratory, University Hospitals Case Medical Center, Cleveland, Ohio, United States of America; 5 Gilead Sciences, Inc., Foster City, California, United States of America; 6 Department of Pathology, Case Western Reserve University, Cleveland, Ohio, United States of America; Centro Nacional de Microbiología - Instituto de Salud Carlos III, Spain

## Abstract

Most studies describing phenotypic resistance to integrase strand transfer inhibitors have analyzed viruses carrying only patient-derived HIV-1 integrase genes (INT-recombinant viruses). However, to date, many of the patients on INSTI-based treatment regimes, such as raltegravir (RAL), elvitegravir (EVG), and dolutegravir (DTG) are infected with multidrug-resistant HIV-1 strains. Here we analyzed the effect of drug resistance mutations in Gag *(*p2/NCp7/p1/p6), protease (PR), reverse transcriptase (RT), and integrase (IN) coding regions on susceptibility to INSTIs and viral replicative fitness using a novel HIV-1 phenotyping assay. Initial characterization based on site-directed mutant INSTI-resistant viruses confirmed the effect of a series of INSTI mutations on reduced susceptibility to EVG and RAL and viral replicative fitness (0.6% to 99% relative to the HIV-1_NL4-3_ control). Two sets of recombinant viruses containing a 3,428-bp *gag*-p2/NCp7/p1/p6/*pol*-PR/RT/IN (p2-INT) or a 1,088 bp integrase (INT) patient-derived fragment were constructed from plasma samples obtained from 27 virologic failure patients participating in a 48-week dose-ranging study of elvitegravir, GS-US-183-0105. A strong correlation was observed when susceptibility to EVG and RAL was assayed using p2-INT- vs. INT-recombinant viruses (Pearson coefficient correlation 0.869 and 0.918, *P*<0.0001 for EVG and RAL, respectively), demonstrating that mutations in the protease and RT have limited effect on susceptibility to these INSTIs. On the other hand, the replicative fitness of viruses harboring drug resistance mutations in PR, RT, and IN was generally impaired compared to viruses carrying only INSTI-resistance mutations. Thus, in the absence of drug pressure, drug resistance mutations in the PR and RT contribute to decrease the replicative fitness of the virus already impaired by mutations in the integrase. The use of recombinant viruses containing most or all HIV-1 regions targeted by antiretroviral drugs might be essential to understand the collective effect of epistatic interactions in multidrug-resistant viruses.

## Introduction

Productive infection with human immunodeficiency virus type 1 (HIV-1) requires three key steps in the replication of the virus, i.e., reverse transcription of viral genomic RNA into viral cDNA by the viral reverse transcriptase (RT), integration of viral cDNA into host cell genome using the viral integrase (IN), and cleavage of newly synthesized viral polypeptide by the viral protease (PR) into individual viral proteins during new virion assembly [1]. Although all three steps were initially considered as targets for antiretroviral drugs, HIV-1 integrase was the last viral enzyme to emerge as a clinically validated alternative to block HIV-1 replication [2]. Since the late 1990s, several different chemical scaffolds have been studied for their ability to inhibit HIV-1 integration [3], [4], leading to the approval of the first HIV-1 integrase strand transfer inhibitor (INSTI) for the treatment of HIV-1 infection in 2007 (raltegravir, RAL, MK-0518, Isentress, Merck Research Laboratories) [5]. Two other promising INSTIs have completed clinical development. Elvitegravir (EVG, JTK-303/GS-9137, Gilead Sciences) [6] has recently been approved in combination with a pharmacokinetic enhancer (cobicistat) and two nucleos(t)ide analog RT inhibitors (emtricitabine and tenofovir) for the treatment of antiretroviral-naïve HIV-infected individuals (QUAD, Stribild, Gilead Sciences) [7]. Dolutegravir (DTG, S/GSK1349572, ViiV Healthcare), a second-generation INSTI [8], recently completed a phase III clinical trial [9] and is waiting approval to treat HIV infection.

Similarly to antiretroviral drugs targeting other steps in the HIV-1 life cycle such as PR, RT, entry, and fusion inhibitors, development of resistance to INSTIs have been documented both *in vitro* and *in vivo* [reviewed in [4], [10], [11]]. Multiple mutations, in at least 26 integrase amino acid positions, have been associated with reduced susceptibility to INSTIs [11]. Resistance to RAL typically evolves through three independent pathways associated with (i) Q148R/H/K, (ii) N155H, or (iii) Y143C/R/H mutations alone or in combination with other IN mutations [4], [10], [11]. *In vitro* selection experiments with EVG identified several INSTI resistance mutations, e.g., H51Y, T66I/A/K, L74M, E92Q/V, Q95K, E138K, S147G, Q148R/K, S153Y/F [12], [13], [14]; however, the most common EVG-resistance mutations that emerged in clinical trials were E92Q, Q148R/H/K, and N155H [15], [16]. Consequently, considerable cross-resistance has been observed between RAL and EVG, mainly related to mutations at codons Q148 and N155 [13], [15], [17]. On the other hand, although reduced susceptibility to DTG has yet to be shown *in vivo* [9], , *in vitro* studies have identified a series of IN mutations following serial virus passages with this INSTI, including H51Y, L101I, G118R, T124A, S153Y/F, and R263K [8], [19]. Moreover, susceptibility to DTG was reduced 8- to 19-fold in site-directed mutant viruses carrying E138K+Q148K, G140S+Q148R, or Q148R+N155H mutations [8] and in viruses obtained from patients failing RAL-containing regimen [20].

Mutations associated with drug resistance generally reduce viral fitness [21], [22], which has been associated with clinical benefits to HIV-infected individuals [23], [24]. The effect of INSTI-resistance mutations on HIV-1 replicative fitness has been better characterized for RAL [25], [26], [27], [28], [29], [30], [31], [32], [33], [34], [35] than for EVG [12], [13], [15], [30], [36], [37] and DTG [19], [38], [39]. Not surprisingly, while most of the primary mutations conferring resistance to INSTIs have a clear negative effect on virus replication, secondary mutations may have either no effect (e.g., S147G), further reduce replication capacity (e.g., V151I), or have a compensatory effect by recovering the fitness of the INSTI-resistant virus (e.g., G140S) [15], [31], [32], [33]. Interestingly, studies evaluating the effect of INSTI-resistance mutations in viral replicative fitness have been based on site-directed mutant viruses [13], [15], [19], [26], [29], [30], [31], [32], [37], [38], IN-recombinant viruses constructed only with patient-derived HIV-1 integrase amplicons [12], [25], [26], [30], [31], [33], or quantifying the dynamics of HIV-1 integrase mutations *in vivo* [27], [34], [40]. Unfortunately, possible interactions among multiple mutations across the HIV-1 genome are difficult to interpret *in vivo* and impossible to study using site-directed mutant or IN-recombinant viruses. Moreover, since INSTIs are being used in both treatment-experienced and treatment-naïve HIV-infected individuals [9], [16], [18], [41], [42], [43], many of these patients may be infected with multidrug-resistant viruses. Therefore, while several studies have shown the effect of mutations outside the protease and the polymerase domain of the RT coding region on susceptibility to PR and RT inhibitors [44], [45], [46], [47], the potential epistatic effects of drug-resistance mutations in the PR and RT coding regions on susceptibility to INSTIs and overall HIV-1 replicative fitness have yet to be fully described [48].

In this study we have used an HIV-1 phenotypic assay (VIRALARTS™HIV), based on the construction of p2-INT (*gag*-p2/NCp7/p1/p6/*pol*-PR/RT/IN) recombinant viruses [49], to (i) characterize the susceptibility of INSTI-resistance viruses to RAL, EVG and twenty additional antiretroviral drugs and (ii) evaluate the role of drug-resistance mutations in PR, RT, and IN coding regions on HIV-1 replicative fitness. We showed that although mutations in PR and RT have a limited effect on susceptibility to INSTIs they affect the ability of the virus to replicate in the presence and absence of RAL and EVG.

## Materials and Methods

### Cells

MT-4 cells (Dr. D. Richman), MT-2 cells (Dr. D. Richman), and U87.CD4.CXCR4 cells (Drs. H. Kui and D. Littman) were obtained from the AIDS Research and Reference Reagent Program, Division of AIDS, NIAID, NIH and the HEK293T cells from Stanford University (Stanford, CA). MT-4 and MT-2 cells were maintained in RPMI 1640/2 mM L-glutamine medium (Cellgro; Mediatech, Herndon, VA) supplemented with 10% fetal bovine serum (FBS; Cellgro), 10 mM N-2-hydroxyethylpiperazine-N-2-ethanesulfonic acid buffer (HEPES; Sigma-Aldrich, St. Louis, MO), 100 U of penicillin/ml, and 100 µg of streptomycin/ml (Gibco; Invitrogen, Carlsbad, CA). U87.CD4.CXCR4 cells were maintained in DMEM medium/L-glutamine (Gibco), 15% FBS (Cellgro), supplemented with 1 µg/ml puromycin, 300 µg/ml G418, and penicillin/streptomycin (Gibco). HEK293T cells were maintained in DMEM medium/L-glutamine (Gibco), 10% FBS (Cellgro), and penicillin/streptomycin (Gibco).

### Antiretroviral Drugs

The antiretroviral drugs used in this study were obtained from the following sources: zidovudine, AZT; didanosine, ddI; stavudine, d4T; lamivudine, 3TC; abacavir, ABC; tenofovir, TDF; emtricitabine, FTC; nevirapine, NVP; delavirdine, DLV; efavirenz, EFV; etravirine, ETR; saquinavir, SQV; ritonavir, RTV; indinavir, IDV; nelfinavir, NFV; amprenavir, APV; lopinavir, LPV; atazanavir, ATV; tipranavir, TPV; and darunavir, DRV (ENZO Life Sciences International, Inc., Plymouth Meeting, PA, formerly BioMol International, LP); raltegravir, RAL and elvitegravir, EVG (Gilead Sciences, Inc., Foster City, CA).

### Construction of Site-directed Mutant (SDM) Viruses Carrying INSTI-associated Mutations

Fourteen SDM viruses containing single, dual- or triple-mutations associated with resistance to INSTI were constructed as previously described [15]. Briefly, the *Apa*1-*Sal*1 fragment of the HIV-1 *pol* gene in vector pUNV5-HisB was mutated using the QuikChange® Site-Directed Mutagenesis Kit (Stratagene; La Jolla, CA) and transformed into PIR1 E. coli cells (Invitrogen; Carlsbad, CA). Plasmid DNA was purified (Qiagen; Valencia, CA), restriction digested with *Apa*1-*Sal*1, and the mutated *pol* fragment ligated to an HXB2 proviral vector and transformed into XL10-Gold *Escherichia coli* cells. Plasmid DNA was then used to construct 3′Gag(p2/NCp7/p1/p6)/PR/RT/INT-recombinant viruses in a HIV-1_NL4-3_ backbone as described below.

### Clinical Specimens

Plasma samples were obtained from twenty-seven patients experiencing virologic failure while participating in a 48-week dose-ranging study of elvitegravir (EVG), Study GS-US-183-0105 [16](Table 1). Written informed consent was obtained from the patients before participation in the study as previously described [15], [16].

**Table 1:** Clinical and virological parameters of 27 HIV-infected individuals participating in the GS-US-183-0105 study of elvitegravir.

			Major Mutations in INT a								EC_50_ FC INSTI		EC_50_ FC PI									EC_50_ FC NNRTI				EC_50_ FC NRTI						
GROUP	Patient	HIV-1 RNA b	E92	N155	Q148	PI (1)	PI (2)	#TAMs	#NAMs	NNRTI (1)	EVG	RAL	APV	TPV	RTV	IDV	NFV	DRV	SQV	LPV	ATV	EFV	NVP	ETR	DLV	AZT	ABC	3TC	FTC	d4T	TDF	ddI
E92Q	08-186	3.96	E92Q			1	5	4	5	2	15	0.5	0.7	0.7	3.7	2.0	MAX	0.3	3.0	1.2	1.0	99	MAX	13	55	10	2.7	1.3	1.1	2.8	2.8	1.3
	08-196	3.81	E92Q			0	4	3	4	2	24	1.4	1.5	0.9	3.3	1.6	25	1.1	2.5	0.7	2.1	MAX	MAX	6.1	15	6.9	5.5	MAX	MAX	2.8	2.8	1.9
E92Q+L68V/I	08-180	5.43	E92Q			8	6	4	6	0	37	1.9	MAX	1.8	MAX	4.5	MAX	MAX	66	MAX	MAX	6.5	4.8	0.7	1.5	40	4.9	MAX	MAX	5.0	5.5	3.8
	08-195	4.65	E92Q			7	4	5	7	3	185	3.7	2.1	0.5	MAX	MAX	MAX	3.6	MAX	MAX	MAX	MAX	MAX	14	56	52	22	MAX	MAX	21	19	24
	08-223	4.25	E92Q			4	9	1	5	0	77	19	MAX	1.1	MAX	MAX	MAX	6.1	MAX	6.4	MAX	3.0	1.6	1.0	2.3	352	17	MAX	MAX	9.4	4.6	9.9
E92Q+N155H	08-202	5.33	E92E/Q	N155N/H		4	2	2	2	1	4.2	1.2	0.8	0.5	5.1	1.5	13	0.7	1.8	0.2	1.3	MAX	MAX	0.3	19	13	2.0	0.6	0.9	3.6	4.9	1.1
	08-230	4.55	E92Q	N155H		4	10	5	7	1	492	18	18	2.7	MAX	MAX	MAX	3.3	MAX	MAX	MAX	65	30	0.5	47	18	9.0	MAX	MAX	3.8	4.4	2.4
E92Q+L68V+N155H	08-210	5.59	E92E/Q	N155N/H		7	5	5	7	3	MAX	6.0	MAX	25	MAX	MAX	MAX	MAX	MAX	MAX	MAX	387	MAX	2.7	113	122	73	MAX	MAX	12	14	13
E92Q+T66I/A	08-198	5.58	E92E/Q			0	3	1	2	0	1.2	1.4	0.9	0.7	3.4	0.6	2.1	0.8	2.7	2.9	2.0	2.8	3.1	0.7	3.1	6.2	1.8	5.1	15	2.9	2.2	0.7
	08-199	3.74	E92E/Q			3	7	5	6	0	59	1.0	MAX	0.8	MAX	4.4	45	83	272	197	102	2.0	1.6	3.0	0.5	76	15	MAX	MAX	5.2	7.0	5.0
N155H	08-245	4.93		N155H		0	2	0	1	0	66	2.4	0.6	0.6	4.7	0.9	1.3	0.9	1.6	0.9	1.0	7.1	3.6	1.1	0.8	3.1	1.9	61	129	3.5	1.2	1.7
	08-240	4.26		N155H		5	6	4	5	0	25	5.2	64	0.5	MAX	5.6	23	27	1.7	MAX	4.8	1.2	2.0	1.0	0.5	61	12	MAX	MAX	14	11	3.0
N155H+S119P/R	08-194	5.66		N155H		3	5	2	4	1	49	2.6	35	2.5	129	4.2	25	2.7	8.8	15	7.8	9.2	5.8	1.5	0.5	5.6	5.5	MAX	MAX	2.9	1.4	3.4
	08-201	5.75		N155H		3	2	0	1	0	128	3.2	2.9	0.9	7.6	2.0	14	1.0	0.9	4.9	1.0	2.6	1.6	1.3	0.5	1.0	3.4	MAX	MAX	1.6	0.8	1.4
N155H+S230R	08-174	4.39		N155H		2	8	5	6	2	53	3.9	84	2.7	MAX	59	MAX	8.7	MAX	176	83	MAX	MAX	43	MAX	23	9.6	MAX	MAX	12	4.9	4.0
Q148R	08-184	3.11			Q148R	0	2	3	4	3	331	10	0.7	0.8	5.0	1.2	10	0.7	3.4	1.0	1.1	MAX	MAX	9.8	101	6.1	8.0	MAX	MAX	5.9	3.3	2.7
GROUP	Patient	HIV-1 RNA b	E92	N155	Q148	PI (1)	PI (2)	#TAMs	#NAMs	NNRTI (1)	EVG	RAL	APV	TPV	RTV	IDV	NFV	DRV	SQV	LPV	ATV	EFV	NVP	ETR	DLV	AZT	ABC	3TC	FTC	d4T	TDF	ddI
Q148R/E138K/S147G	08-182	5.50			Q148R	3	0	0	3	0	179	5.4	66	0.6	8.8	1.1	7.7	7.8	3.8	7.8	9.2	MAX	MAX	293	133	1.9	13	MAX	MAX	7.2	2.9	8.4
	08-209	4.57			Q148R	6	6	0	8	2	219	8.9	MAX	3.1	MAX	29	MAX	MAX	MAX	MAX	MAX	MAX	MAX	54	264	43	65	MAX	MAX	16	3.3	21
Q148R/H/K+G140S/C	08-197	3.36			Q148R	4	4	4	6	2	MAX	13	MAX	16	MAX	328	MAX	68	MAX	9.6	450	458	156	6.9	8.2	74	14	MAX	MAX	43	8.8	7.9
	08-239	4.45			Q148H	6	8	5	6	1	MAX	113	125	24	MAX	63	MAX	58	82	MAX	MAX	115	85	12	6.2	82	17	MAX	133	13	6.9	3.0
Q148R/H/K+N155H	08-205	4.20		N155N/H	Q148Q/R	4	3	5	6	1	144	5.1	46	5.7	MAX	MAX	MAX	22	MAX	MAX	MAX	31	26	0.8	1.4	79	7.3	1.6	1.6	4.5	9.2	1.9
	08-232	3.81		N155N/H	Q148Q/R	5	7	5	6	1	788	41	MAX	62	MAX	MAX	MAX	48	7.5	MAX	46	126	238	1.0	33	159	34	MAX	MAX	33	19	3.9
T66I/A+other mutations	08-175	4.75				5	5	5	7	2	17	0.9	17	0.8	MAX	7.6	34	6.0	67	32	44	MAX	133	6.5	210	4.2	12	MAX	MAX	4.0	1.5	2.2
	08-193	5.37				4	5	5	6	0	23	0.9	4.8	2.9	MAX	1.2	19	1.1	5.8	8.0	8.7	1.9	6.5	0.7	0.5	14	7.1	MAX	MAX	4.4	3.0	1.7
Other INSTI-R (no 66, 92, 148, 155)	08-200	3.72				5	5	1	6	2	28	2.2	9.4	21	MAX	9.8	122	5.0	10	57	66	MAX	MAX	1.9	MAX	19	12	MAX	MAX	17	2.6	5.9
	08-236	3.83				6	6	5	7	2	47	0.9	MAX	4.4	MAX	56	253	96	179	172	MAX	181	MAX	1.2	MAX	15	9.4	MAX	MAX	4.5	2.7	2.3
No INSTI-R mutations	08-179	4.43				6	6	5	6	1	0.7	0.6	17	8.8	MAX	16	MAX	2.7	96	106	81	MAX	MAX	0.8	0.6	38	15	169	112	9.0	7.2	3.2

a Major mutations associated with resistance to INSTI as described [4], [11].

b Plasma HIV viral load (log10 copies/ml).

INSTI-R, mutations associated with resistance to INSTI; PI (1), number of primary mutations associated with resistance to PI; PI (2), number of secondary mutations associated with resistance to PI; #TAMs, number of thymidine analogue-associated mutations; #NAMs, number of nucleoside analogue-associated mutations; NNRTI (1), number of primary mutations associated with resistance to NNRTI. EC_50_ FC, based on VIRALARTS™HIV three independent EC_50_ replicates for each drug were used to calculate the fold changes (FC) of the query viruses relative to the HIV-1_NL4-3_ control and the mean EC_50_ FC is indicated. MAX, complete virus inhibition was not achieved using the maximum drug concentration, i.e., virus was completely resistant to the respective antiretroviral drug.

### RT-PCR Amplification and Nucleotide Sequence Analysis of *gag*-p2/NCp7/p1/p6/*pol*-PR/RT/IN-coding Sequences

Plasma viral RNA was purified from pelleted virus particles by centrifuging one milliliter of plasma at 14,000g×60 min at 4°C, removing 860 µl of cell-free supernatant and resuspending the pellet in the remaining 140 µl, to finally extract viral RNA using QIAamp Viral RNA Mini kit (Qiagen; Valencia, CA). Viral RNA was reverse-transcribed using AccuScript High Fidelity Reverse Transcriptase (Stratagene Agilent; Santa Clara, CA) and the corresponding antisense external primer in 20 µl reaction mixture containing 1 mM dNTPs, 10 mM DTT and 10 units of RNAse inhibitor. A 3,428 nt HIV-1 genomic region encoding the Gag proteins p2, p7, p1 and p6, and the protease, reverse transcriptase, and integrase enzymes was then PCR amplified using a series of external and nested primers with defined cycling conditions [49]. External PCR reactions were carried out in a 50-µl mixture containing 0.2 mM dNTPs, 2.5 mM MgCl_2_ and 2.5 units of Pfu Turbo DNA Polymerase (Stratagene). Nested PCR reactions were carried out in 50-µl mixture containing 0.2 mM dNTPs, 0.3 units of Pfu Turbo DNA Polymerase and 1.9 units of Taq Polymerase (Denville Scientific; Metuchen, NJ). PCR products corresponding to the *gag*-p2/NCp7/p1/p6/*pol*-PR/RT/IN-coding region of HIV-1 were purified with the QIAquick PCR Purification kit (Qiagen) and sequenced using AP Biotech DYEnamic ET Terminator cycle with Thermosequenase II (Davis Sequencing LCC, Davis, CA). Nucleotide sequences were analyzed using DNASTAR Lasergene Software Suite v.7.1.0 (Madison, WI).

### Construction of *gag*-p2/NCp7/p1/p6/*pol*-PR/RT/IN - and INT-recombinant Viruses

**Figure 1:**
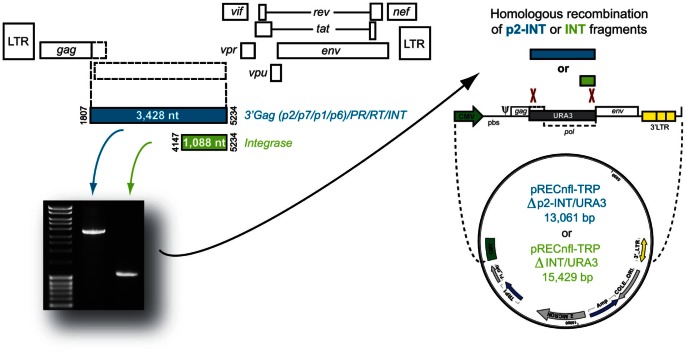
Strategy to introduce patient-derived *gag*-p2/NCp7/p1/p6/*pol*-PR/RT/IN or IN PCR fragments into a proprietary vector via yeast homologous recombination as described [49].

Two sets of infectious recombinant viruses were constructed from each clinical specimen in a HIV-1_NL4-3_ backbone using a novel yeast-based cloning technology as described [49]. Briefly, PCR products spanning the 3′ end of *gag* (p2/p7/p1/p6) and the entire *pol* gene (PR/RT/IN; p2-INT; 3,428 nt) or the integrase-coding region only (INT; 1,088-nt) were introduced via yeast homologous recombination into pRECnfl-TRP?p2-INT/URA3 or pRECnfl-TRP?INT/URA3 vectors, respectively, containing a near-full length HIV-1 genome with the yeast uracil biosynthesis (URA3) gene replacing the respective p2-INT or INT HIV-1 coding sequences (Fig. 1). Following yeast transformation, vector DNA was purified from the entire number of yeast colonies (typically 200 to 500 individual colonies) and used to transform Electrocomp TOP10 bacteria (Invitrogen). Plasmid DNA from the entire bacteria preparation – to guarantee the continuity of the viral population that may have existed in vivo – was purified from 10 ml of bacteria culture (QIAprep Spin Miniprep Kit, Qiagen) and used to introduce the patient-derived HIV-1 sequences into a pNL4-3-hRluc vector expressing the human Renilla luciferase gene (hRluc) [50] as described [49]. Four micrograms of the resulting plasmid were transfected into HEK293T cells using GenDrill™ (BamaGen® Bioscience; Gaithersburg, MD). Cell culture supernatant was harvested 48 hours post-transfection, clarified by centrifugation at 700×g, filtered through a 0.45 µm steriflip filter (Millipore; Billerica, MA), aliquoted, and stored at −80°C until further use. Tissue culture dose for 50% infectivity (TCID_50_) was determined in triplicate for each serially diluted virus using the Reed and Muench method [51] and viral titers expressed as infectious units per milliliter (IU/ml). The p2-INT and INT regions from the p2-INT- and INT-recombinant viruses, respectively, were sequenced as described above to verify the identity and genotype of each constructed virus.

### Drug Susceptibility Based in an MT-2 Assay

Susceptibility of the fourteen SDM viruses to EVG and RAL was quantified using cell-viability of single viral infections [15]. Briefly, the multiplicity of infection (MOI) of the viruses was normalized to obtain a signal-to-noise ratio of 4 to 7 for uninfected versus infected cells in the absence of drug (chemiluminescent detection, Cell Titer-Glo, Promega). MT-2 cells were infected for three hours then incubated in triplicate drug dilution series for five days at 37°C. Cell viability data was converted to percentage of cell death and drug concentrations required to inhibit virus replication by 50% (EC_50_) were calculated by curve fitting (GraphPad Prism v.5.01, GraphPad Software, La Jolla, CA). Fold change (FC) resistance values were calculated by dividing the mean EC_50_ of the query virus (HIV-1_SDM_) by the mean EC_50_ of the control (HIV-1_HXB2_).

### Drug Susceptibility Determination Using VIRALARTS™HIV

Drug susceptibility of fourteen SDM viruses, twenty-seven p2-INT-, and twenty-seven INT-recombinant viruses was measured by determining the extent to which antiretroviral drugs inhibited viral replication in MT-4 cells (and in U87.CD4.CXCR4 cells in the case of the SDM viruses) as described [49]. Briefly, serial dilutions spanning empirically determined ranges of each drug were added in triplicate in 96-well plates in RPMI medium with L-glutamine (Cellgro; Mediatech) supplemented with 10% fetal bovine serum, 100 U of penicillin/mL, 100 µg of streptomycin/mL, (Mediatech) and 10 mM HEPES (Sigma-Aldrich) for MT-4 cells or DMEM medium/L-glutamine (Gibco), 10% FBS (Cellgro), supplemented with 1 µg/ml puromycin, 300 µg/ml G418, and penicillin/streptomycin (Gibco) for U87.CD4.CXCR4 cells. MT-4 cells were infected with either the reference virus (HIV-1_NL4-3-hRluc_) [50] or the corresponding query virus (HIV-1_SDM- or p2-INT- or INT-hRluc_) expressing human Renilla luciferase at a multiplicity of infection (MOI) of 0.005 IU/cell for one hour at 37°C, 5% CO_2_. HIV-infected MT-4 cells were then resuspended in RPMI medium and 30,000 cells were added to each well containing pre-plated antiretroviral drugs. In the case of U87.CD4.CXCR4 cells, serial dilutions of the antiretroviral drugs were added to 5,000 cells/well two hours prior infection with either the reference virus (HIV-1_NL4-3-hRluc_) or the corresponding query virus (HIV-1_SDM- or p2-INT- or INT-hRluc_) at a MOI of 0.005 IU/cell. Virus replication was quantified 72 or 120 hours post-infection for MT-4 and U87.CD4.CXCR4, cells respectively, by measuring renilla luciferase activity (relative light units, RLU) using the *Renilla* Luciferase Assay System (Promega, Madison, WI) in a multiwell plate reader (Victor V multilabel reader, PerkinElmer, Waltham, MA). Drug concentrations required to inhibit virus replication by 50% (EC_50_) were calculated by (i) plotting the percent inhibition of luciferase activity versus log_10_ drug concentration and (ii) fitting the inhibition curves to the data using nonlinear regression analysis (GraphPad Prism v.5.01, GraphPad Software, La Jolla, CA). Fold change (FC) resistance values were calculated by dividing the mean EC_50_ of the query virus (HIV-1_SDM- or p2-INT- or INT-hRluc_) by the mean EC_50_ of the internal control (HIV-1_NL4-3-hRluc_) in each assay.

### HIV-1 Replicative Fitness Determination Using Viral Growth Kinetics Analysis

The ability of fourteen SDM viruses, twenty-seven p2-INT-, and twenty-seven INT-recombinant viruses, plus the HIV-1_NL4-3_ wild-type control, to replicate in the absence or presence of drug pressure (EVG or RAL) was determined by measuring viral growth kinetics as described [49], [52]. Briefly, 3×10^6^ MT-4 cells were infected in triplicate at a MOI of 0.01 IU/cell in one ml of culture medium and incubated for 2 hrs at 37°C in 5% CO_2_. HIV-infected cells were then washed two times with 1× PBS, then split to be cultured in triplicate wells of a 24-well plate (1×10^6^ cells/well). Culture supernatant was assayed using a reverse transcriptase assay on days 0, 3, 4, 5, 6, 7, and 10 post-infection as described [53]. Viral replication was quantified using the slope of the growth curves and performing linear regression analysis derived from the equation *log(y) = mt+log(h*), where *y* is virus quantity (cpm), *t* is time in days, and *h* is the *y*-intercept (day 0). All slope values for each virus were used to calculate the mean, standard deviation, and 10^th^ & 90^th^ percentiles. Differences in the mean values were evaluated using a One Way Analysis of Variance test and the significance difference from the reference HIV-1_NL4-3_ virus calculated using the Bonferroni’s Multiple Comparison Test (GraphPad Prism v.5.01, GraphPad Software).

### HIV-1 Replicative Fitness Determination Using Growth Competition Experiments

Dual infection/competition experiments were carried out as previously described [52], [53], [54]. Briefly, query (HIV-1_SDM- or p2-INT- or INT_) and control (HIV-1_NL4-3_) viruses tagged with hRluc and fluc2, respectively, were competed in a 1∶1 initial proportion using a MOI of 0.01 IU/cell. One ml of the virus mixture was incubated with 6×10^5^ MT-4 cells for 2 h at 37°C, 5% CO_2_ in the presence and absence of 0.01 nM EVG or 1 nM RAL, corresponding to the EC_50_ values for these INSTIs using HIV-1_NL4-3_ as determined by VIRALARTS™HIV. Cells were subsequently washed three times with 1× PBS and cultured in triplicate in a 96-well plate (2×10^5^ cells/well) with the correspondent amount of drug. At day 5 post-infection, the final proportion of the two viruses in each competition was quantified using the Dual-Glo® Luciferase Assay System (Promega, Madison, WI) in a multiwell plate reader (Victor V°multilabel reader, PerkinElmer) after normalizing to viral production in the HIV-1 monoinfections as described [53], [54], [55]. Replicative fitness for each virus was calculated and expressed as a percentage of the replicative fitness of the reference virus HIV-1_NL4-3_.

### Statistical Analyses

Descriptive results are expressed as median values and interquartile ranges. Pearson correlation coefficient was used to determine the strength of association between categorical variables. All differences with a *P* value of <0.05 were considered statistically significant. As described above, differences in the mean of the slope values for the viral growth kinetics curves were determined using a One Way Analysis of Variance test and the significance difference from the reference HIV-1_NL4-3_ virus calculated using the Bonferroni’s Multiple Comparison Test. All statistical analyses were performed using GraphPad Prism v.5.01 (GraphPad Software, La Jolla, CA) unless otherwise specified.

## Results

### Susceptibility to EVG and RAL of p2-INT-recombinant Viruses Carrying Mutations Associated with Resistance to INSTIs

**Figure 2:**
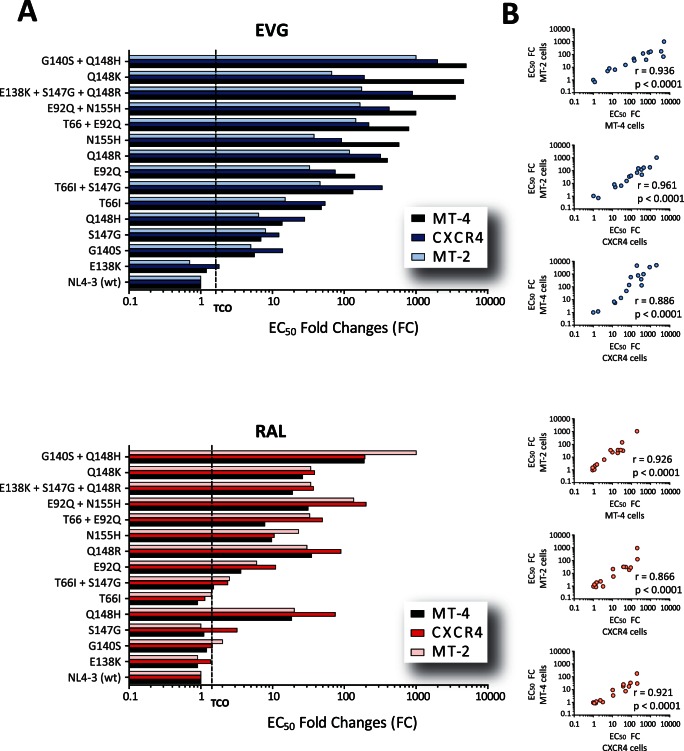
Susceptibility to EVG and RAL of 14 p2-INT-recombinant viruses carrying mutations associated with resistance to INSTIs. (A) Drug susceptibility evaluated by measuring luciferase expression in triplicate in MT-4 cells (VIRALARTS™HIV) and CXCR4 cells, and cell viability in MT-2 cells. Technical cut-off (TCO) values for EVG (1.62-fold) and RAL (1.42-fold) were calculated by repeatedly testing the reference HIV-1_NL4-3_ strain using VIRALARTS™HIV. (B) Pearson correlation coefficient was used to determine the strength of association between the EC_50_ fold changes calculated using the three drug susceptibility assays. r, correlation coefficient and p, two-tailed p value.

We recently developed an HIV-1 phenotyping assay (VIRALARTS™HIV) based on the introduction of patient-derived p2-INT amplicons into an HIV-1_NL4-3_ backbone using a yeast-based cloning system [49], [56]. This assay was validated using 21 antiretroviral drugs, including RAL [49]; however, EVG was not part of the original characterization. Thus, here we first tested the ability of VIRALARTS™HIV to quantify susceptibility to EVG and RAL by comparing our results with data obtained with an MT-2 assay. For that, p2-INT fragments were PCR amplified from 14 plasmids containing single, dual or triple INSTI resistance mutations introduced by site-directed mutagenesis, and then used to construct p2-INT-recombinant viruses. Drug susceptibility was assessed by measuring luciferase expression in MT-4 cells (original VIRALARTS™HIV) and CXCR4 cells, and cell viability in MT-2 cells. Overall, susceptibility to EVG and RAL was similar in all three cell systems (Fig. 2A), with strong statistically significant correlations (*r* values ranging from 0.866 to 0.961, *P*<0.0001, Pearson coefficient correlation), particularly between data obtained with MT-4 and MT-2 cells (Fig. 2B). As expected, a few mutations conferred resistant to EVG but retained susceptibility to RAL, e.g., T66I, G140S, and S147G, while other mutations reduced susceptibility to both INSTIs, e.g., Q148K/R/H, N155H (Fig. 2A). All viruses were susceptible to d4T and NVP, antiretroviral drugs used as controls (data not shown). Interestingly, the wild type reference control virus (HIV-1_NL4-3_) was particularly susceptible to EVG using MT-4 cells in this system (EC_50_ values ranging from 0.009 to 0.02 nM, data not shown). Thus, any reduction in susceptibility due to the presence of INSTI resistance mutations seems to be magnified in this system producing higher than previously reported EC_50_ fold-change values (Fig. 2A).

### Replicative Fitness of p2-INT-recombinant Viruses Carrying Mutations Associated with Resistance to INSTIs in the Absence and Presence of EVG or RAL

**Figure 3:**
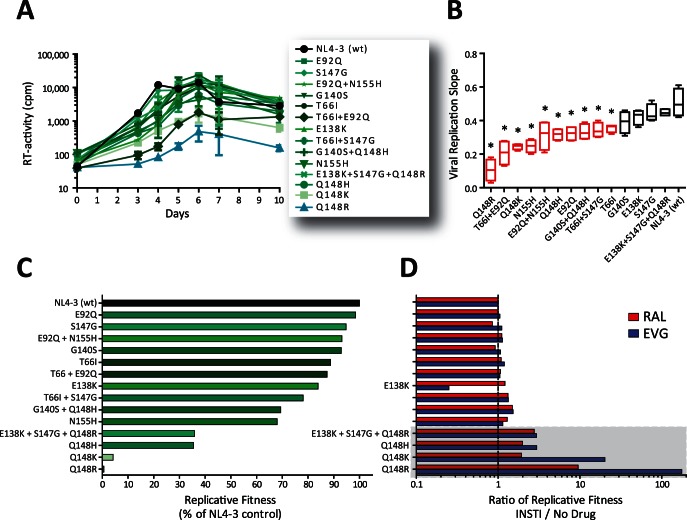
Replicative fitness of p2-INT-recombinant viruses carrying mutations associated with resistance to INSTIs in the absence and presence of EVG or RAL. (A) Fifteen p2-INT-recombinant viruses (i.e., 14 INSTI-resistance and the HIV-1_NL4-3_ wild-type virus) were evaluated for their ability to replicate in MT-4 cells in the absence of drug pressure. Virus replication was quantified by measuring reverse transcriptase (RT) activity in the cell-free supernatant. Error bars indicate the range of values obtained from three independent experiments. (B) Viral replication slopes were calculated using the slopes between RLU values at days 0 & 3, 0 & 4, 0 & 5, and 0 & 6. All four slope values for each virus were used to calculate the mean, standard deviation, and 10^th^ & 90^th^ percentiles. Differences in the mean values were calculated using a One Way Analysis of Variance test and the significance difference from HIV-1_NL4-3_ calculated using the Bonferroni’s Multiple Comparison Test. The replication kinetics of viruses marked with an asterisk (*) were significantly different from the HIV-1_NL4-3_ control (p<0.05, 95% CI). Each p2-INT-recombinant virus was competed against the HIV-1_NL4-3_ control in the absence (C) or presence (D) of EVG (0.01 nM) or RAL (1 nM) and their replicative fitness calculated and expressed as a percentage of the replicative fitness of the reference virus (HIV-1_NL4-3_) as described [55], [69]. The Ratio of Replicative Fitness was calculated dividing the % Replicative Fitness in the presence of drug (EVG or RAL) by the % Replicative Fitness in the absence of drug pressure; e.g., 105.5% ÷ 0.6% = 176x, for Q148R. Values represent results obtained from single growth competition experiments.

One of the advantages of VIRALARTS™HIV is that, unlike other HIV-1 phenotyping assays based in single-cycle replication, it produces replication-competent p2-INT-recombinant viruses and supports the analysis of replication kinetics during multiple rounds of cell-culture infections. Here we used two different but complementary approaches to quantify the ability of the 14 p2-INT-recombinant viruses, carrying single, dual or triple INSTI resistance mutations to replicate in the presence and absence of EVG and RAL. A first glimpse of the replicative fitness of these viruses was obtained using classical viral growth kinetics in MT-4 cells in the absence of drug as compared to the reference HIV-1_NL4-3_ strain (Fig. 3A). Statistical analysis of the slope of the growth curves showed that the replicate fitness of viruses containing the single mutations E138K, S147G, and G140S and the triple mutant E138K+S47G+Q148R was similar to the wild type HIV-1_NL4-3_ (Fig. 3B). On the other hand, 10 INSTI-resistant viruses showed an impairment in replication compared to the wild type control, with the Q148R virus being the less fit (Fig. 3B).

Since *in vitro* growth competition experiments are considered the gold standard method to measure viral fitness [21], [22] we used dual infections to quantify the ability of the 14 INSTI-resistant viruses to replicate in the absence and presence of drug pressure (EVG and RAL). Viruses carrying the Q148R or Q148K single mutations showed a marked decrease in replicative fitness, i.e., 0.6% and 4.2% relative to that of the HIV-1_NL4-3_ control virus, respectively (Fig. 3C). Interestingly, the virus with a different positive charged amino acid in the same position (Q148H) had a 9- to 60-fold higher replicative fitness than either the Q148R or Q148K viruses (36% of the HIV-1_NL4-3_, Fig. 3C). Moreover, mutations E138K (84%) and S147G (95%), which seem to have a minimal effect on INSTI resistance (Fig. 2A) and viral fitness contributed to increase the effect of the Q148R mutation, as observed in the triple mutant E138K+S147G+Q148R (36% of the HIV-1_NL4-3_, Fig. 3C). The rest of the p2-INT viruses carrying single or double INSTI resistance mutations showed a range of replicative fitness values from 68% (N155H) to 99% (E92Q) relative to the HIV-1_NL4-3_ control (Fig. 3C). No statistically significant correlation was observed between the viral replication slope values (viral growth kinetics) and the % replicative fitness calculated using growth competition (data not shown), driven mainly by discrepancies with the results from viruses E92Q, E92Q+N155H, and E138K+S147G+Q148R (Figs. 3B and 3C).

Finally, each of the 14 INSTI-resistant viruses was competed against the HIV-1_NL4-3_ control virus in the presence of EVG (0.01 nM) or RAL (1 nM). The ratio between the replicative fitness of the virus in the presence and absence of drug pressure serves as an indirect measurement of the level of resistance, and the effect on virus replication, provided by certain mutation(s). For example, a ratio of 1 indicates that the mutant virus replicates similarly to the wild type control virus both in the presence and absence of drug pressure (i.e., mutation has a small effect in replicative fitness); however, a positive ratio underlines the ability of the virus to replicate in the presence of the drug and/or the detrimental effect of the mutation(s) on fitness in the absence of drug pressure. Accordingly, INSTI-resistant viruses with replicative fitness values ranging from 68% to 99% of the HIV-1_NL4-3_ control in the absence of drug had ratios of drug/no drug replicative fitness in the range of 0.9× to 1.5× (Fig. 3D). The ratio increased to 2× and 3× for viruses carrying mutations Q148H/K and E138K+S147G+Q148R, both with a replicative fitness value of 36%. The two viruses with the lowest replicative fitness in the absence of drug, i.e., Q148K (4.2%) and Q148R (0.6%) showed a substantial recovery on replication capacity in the presence of the INSTIs with ratios of drug/no drug replicative fitness ranging from 10× to 176×, respectively (Fig. 3D). Interestingly, the ratio of replicative fitness of the E138K virus, susceptible to both INSTIs, was reduced in the presence of EVG (0.3×) but not with RAL (1.2×, Fig. 3D).

### Antiretroviral Drug Susceptibility of Multidrug-resistant Viruses Obtained from Patients Participating in a Phase II Clinical Trial of Elvitegravir

Patient-derived PCR products from 27 HIV-infected individuals participating in a 48-week dose-ranging study of elvitegravir, GS-US-183-0105 [16] were used to construct p2-INT-recombinant viruses and their susceptibility to 22 antiretroviral drugs, including EVG and RAL, was assessed using VIRALARTS™HIV [49]. Viruses were divided in groups based on the presence of amino acid substitutions in three positions associated with major INSTI mutations (i.e., E92, N155, and Q148) and secondary mutations (Table 1). Table S1 includes a full list of amino acid substitutions in the PR, RT, and IN coding regions obtained by population sequencing of plasma samples. As expected from highly treatment-experienced HIV-infected individuals, most of them carried viruses with multiple primary mutations conferring resistance to many protease, RT, and IN inhibitors. These HIV-1 genotypes were corroborated by the drug susceptibility assay and in the case of INSTIs, mutations Q148R/H, N155H+Q148R, and E92Q+N155H conferred the highest level of resistance to EVG and RAL (Table 1).

### Comparison of the Susceptibility to EVG and RAL between Multidrug-resistant p2-INT- and INT-recombinant Viruses

**Figure 4:**
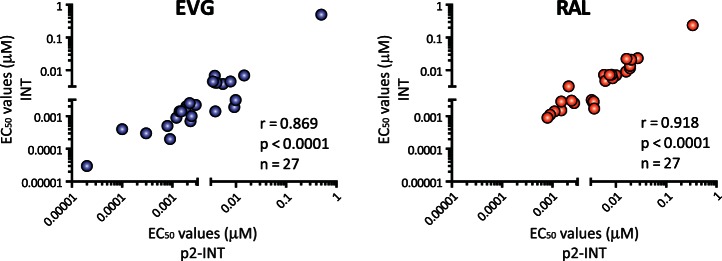
Comparing susceptibility to EVG and RAL using p2-INT- and INT-recombinant viruses. Patient-derived PCR products from 27 highly treatment-experienced individuals participating in the GS-US-183-0105 study of elvitegravir were used to construct p2-INT or INT-recombinant viruses and their susceptibility to EVG and RAL determined using VIRALARTS™HIV in triplicate. Pearson correlation coefficient was used to determine the strength of association between the EC_50_ values calculated using the two sets of recombinant viruses. r, correlation coefficient; p, two-tailed p value; and n, number of viruses analyzed per drug.

As described above, to date most of the INSTI susceptibility data has been obtained using INT-recombinant viruses constructed only with patient-derived HIV-1 integrase amplicons [12], [25], [26], [30], [31], [33]. Since our HIV-1 phenotyping assay uses recombinant viruses carrying longer patient-derived amplicons (*gag*-p2/NCp7/p1/p6/*pol*-PR/RT/IN) we compared the susceptibility to EVG and RAL using both p2-INT- and INT-recombinant viruses, with the first set of viruses carrying the 3′end of Gag, PR, and RT coding regions, in addition to the integrase, from the 27 patients (Fig. 1). A strong statistically correlation was observed between the EC_50_ values calculated with both sets of viruses, i.e., *r* values of 0.869 and 0.918 (*P*<0.0001, Pearson coefficient correlation) for EVG and RAL, respectively (Fig. 4).

### Replicative Fitness of Multidrug-resistant p2-INT- and INT-recombinant Viruses in the Absence and Presence of EVG or RAL

**Figure 5:**
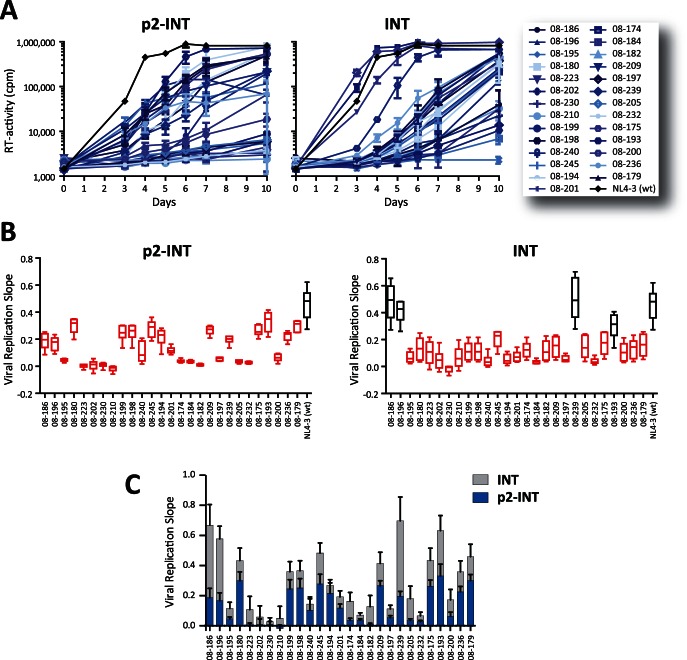
Viral growth kinetics of multidrug-resistant p2-INT- and INT-recombinant viruses in the absence of drug pressure. (A) Twenty-eight p2-INT-recombinant viruses (i.e., 27 INSTI-resistance and the HIV-1_NL4-3_ wild-type virus) were evaluated for their ability to replicate in MT-4 cells in the absence of drug pressure. Virus replication was quantified by measuring reverse transcriptase (RT) activity in the cell-free supernatant. Error bars indicate the range of values obtained from three independent experiments. (B) Viral replication slopes were calculated using the slopes between RLU values as described in the legend of Figure 3. (C) Comparison of the mean viral replication slope between p2-INT and INT-recombinant viruses constructed from the same HIV-infected individual.

Multiple drug-resistance mutations in the PR and RT coding regions have been associated with impairing the ability of the virus to replicate in the absence of drug pressure [21], [22]. Thus, although susceptibility to INSTI may not be affected by mutations in the PR and RT (Fig. 4), detrimental mutations in these HIV-1 genomic regions may work together with mutations in the integrase to affect the overall replicative fitness of the virus. We first compared the viral growth kinetics of the p2-INT- and INT-recombinant viruses in the absence of drug (Fig. 5A). All 27 p2-INT-recombinant viruses, many of them harboring numerous mutations in the PR, RT, and IN coding regions (Table 1 and Table S1), showed different levels of impairment on their replication kinetics compared to the HIV-1_NL4-3_ control (Fig. 5B). In contrast, not all INT-recombinant viruses, constructed only from patient-derived HIV-1 IN amplicons, showed a decrease in replicative fitness. The replication kinetics of viruses from patients 08-186 (E92Q), 08-196 (E92Q), 08-239 (G140S+Q148H), and 08-193 (T66A) was not different to that of the wild type control virus (Fig. 5B). Moreover, while the rest of the INT-recombinant viruses showed a decrease in replicative fitness, most of their viral replication slopes were higher than those calculated for the p2-INT-recombinant (Fig. 5C).

**Figure 6:**
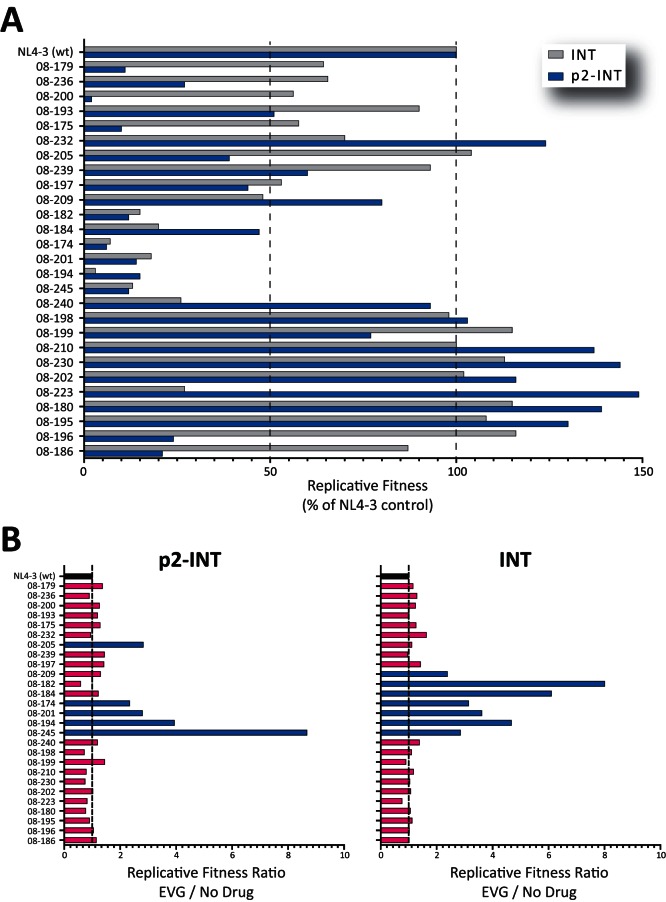
Growth competition experiments to determine replicative fitness of multidrug-resistant p2-INT- and INT-recombinant viruses in the absence and presence of drug pressure. p2-INT- and INT-recombinant viruses were constructed using patient-derived PCR products from 27 highly treatment-experienced individuals participating in the GS-US-183-0105 study of elvitegravir. Each recombinant virus was competed against the HIV-1_NL4-3_ control in the absence (A) or presence (B) of EVG (0.01 nM) and their replicative fitness calculated and expressed as a percentage of the replicative fitness of the reference virus (HIV-1_NL4-3_) as described [55], [69]. Values represent results obtained from single growth competition experiments. The Ratio of Replicative Fitness was calculated as described in the legend of Figure 3.

As with the INSTI-resistant viruses constructed from site-directed mutants, we used growth competition experiments to determine the ability of the p2-INT and INT-recombinant viruses to replicate in the absence and presence of drug pressure, in this case EVG. A variety of replicative fitness values were observed in both sets of viruses, ranging from 2% to 149% (p2-INT-recombinant viruses) and 4% to 116% (INT-recombinant viruses) relative to the HIV-1_NL4-3_ control, in the absence of drug pressure (Fig. 6A). A marked reduction in replicative fitness (below 50%) was observed in 15/27 and 9/27 p2-INT- and INT-recombinant viruses, respectively; mostly related to viruses carrying mutations at positions Q148 and N155 in the integrase coding region (Fig. 6A). Differences in replicative fitness between p2-INT- and INT-recombinant viruses were patient-dependent and guided mainly by the number and type of drug resistance mutations in the PR and RT coding regions (Table 1 and Table S1). Finally, only viruses with highly impaired replicative fitness in the absence of drug pressure (e.g., those carrying N155H and/or Q148R mutations) showed a significant recovery of fitness in the presence of EVG, particularly using INT-recombinant viruses (Fig. 6B). Moreover, changes in viral replicative fitness in the presence of EVG correlated with the level of resistance (EC_50_ fold changes) to EVG using INT-recombinant viruses (*r* = 0.433, *P* = 0.02) but not with p2-INT-recombinant viruses (*r* = 0.145, *P* = 0.46, data not shown).

## Discussion

Multiple studies have analyzed the role of resistance mutations in the HIV-1 integrase coding region on susceptibility to INSTI and their effect on viral replication capacity [3], [4], [10], [11]. However, all these studies have used site-directed mutant viruses [13], [15], [19], [26], [29], [30], [31], [32], [37], [38], IN-recombinant viruses based only on patient-derived HIV-1 integrase amplicons [12], [25], [26], [30], [31], [33], or quantified the dynamics of HIV-1 integrase mutations *in vivo* [27], [34], [40]. Here we used a novel system to construct recombinant viruses carrying patient-derived HIV-1 fragments covering all the main regions in the *pol* gene, which is targeted by 24 of the 26 antiretroviral drugs approved for the treatment of HIV infection [49]. These p2-INT (*gag*-p2/NCp7/p1/p6/*pol*-PR/RT/IN)-recombinant viruses allowed us to characterize the susceptibility of INSTI-resistant viruses to twenty-two antiretroviral drugs, including RAL and EVG, and evaluate the role of drug-resistance mutations in PR, RT, and IN coding regions on HIV-1 replicative fitness.

Three main pathways of resistance to RAL have been associated with primary mutations Q148R/H/K, N155H, or Y143C/R/H, either alone or in combination with other IN mutations [3], [11]. EVG can select several INSTI resistance mutations *in vitro* [12], [13], [14] but E92Q, Q148R/H/K, and N155H mutations have been the most common EVG resistance mutations identified *in vivo*, with other mutations also emerging during clinical studies, e.g., T66I/A/K, S147G, etc. [4]. Therefore, we first tested our system by constructing p2-INT-recombinant viruses carrying these and other single, dual, or triple INSTI resistance mutations introduced by site-directed mutagenesis [15]. Susceptibility to EVG and RAL quantified with our HIV-1 phenotyping assay (VIRALARTS™HIV) correlated with results from a standard drug resistance test based on MT-2 cells, with mutations Q148H/K/R and N155H conferring the highest level of resistance to both RAL and EVG. This is in agreement with previous reports of cross-resistance between these two INSTIs, mainly related to mutations at these two positions [3], [4], [10], [11]. In addition, since VIRALARTS™HIV is a multiple replication cycle assay, we were able to study the effect of these mutations on viral replication fitness by measuring viral growth kinetics in single infections or growth competition experiments. Nearly all amino acid substitutions associated with INSTI resistance had an effect on HIV-1 replicative fitness. As previously described [29], [31], [33], [36], [37], mutations associated with higher loss of susceptibility to INSTIs, such as Q148H/R/K and N155H had a major effect on the ability of the virus to replicate in the absence of drug pressure. Moreover, only the highly resistant viruses with low replicative fitness in the absence of drug (i.e., Q148R, Q148K, Q148H, and E138K+S147G+Q148R) showed a significant increase in their capacity to replicate in the presence of INSTIs. It is possible that higher INSTI concentrations (i.e., >EC_50_ values) could lead to a more marked recovery in fitness for viruses with moderate resistance to INSTI (e.g., T66I, E92Q, or G140S). In summary, these experiments not only corroborated previous studies but also validated our assay to be used with patient-derived HIV-1 fragments to evaluate the potential epistatic relationship between drug resistance mutations in the HIV-1 *pol* gene.

Interestingly, although most of the replicative fitness values determined by viral growth kinetics or growth competition experiments shared the same trend, we were not able to calculate a statistically significant correlation between the fitness estimated by these two complementary methodologies. In the case of the p2-INT-recombinant viruses constructed from the site-directed mutants, the discrepancies could be attributed to three particular viruses: E92Q, E92Q+N155H, and E138K+S147G+Q148R. It is possible that (i) intrinsic differences between the two tests, (ii) the method used to determine TCID_50_ values, and/or (iii) the fact viral growth kinetics were performed in triplicate while growth competitions experiments were performed only once, could have contributed to the differences in fitness observed for some viruses. Nevertheless, and due to the fact that *in vitro* growth competition experiments are considered the gold standard method to measure viral fitness [21], [22], here we believe that the data generated by the competitions is more reliable than the results obtained with viral growth kinetics.

The role of drug resistance mutations in the PR and RT coding regions on susceptibility to INSTIs and their contribution, together with INSTI resistance mutations, to overall HIV-1 replicative fitness is not fully understood. Drug susceptibility studies of protease and RT inhibitors are usually performed separately from those evaluating resistance to INSTIs, typically by constructing independent PR/RT- and IN-recombinant viruses, respectively. This makes difficult, if not impossible, to analyze the natural interaction among drug resistance mutations in the three HIV-1 enzymes. For example, several studies have shown that mutations outside the protease and the polymerase domain of the RT coding region have an effect on susceptibility to PIs and RTIs, respectively. Mutations downstream of the Gag protease cleavage site p24(CA)/p2 have been associated with reduced susceptibility to PIs [44], [57], while amino acid substitutions in the connection [46], [58] and RNase H [59] domains of the RT have been shown to have an effect on NRTI and NNRTI resistance. In addition, HIV-1 integrase seems to affect nuclear import and viral maturation while its interaction with the RT may affect virus replication [60]. More important for this study, a few specific polymorphisms in the integrase-coding region (e.g., M154L, V165I, G163R, and T206S) seem to be associated with RT resistance mutations in antiretroviral-experienced individuals [61]. Thus, it is only logic to predict that drug resistance mutations in PR, RT, and IN play a role in overall HIV-1 replicative fitness, perhaps affecting the ability of the virus to respond to certain combination(s) of antiretroviral drugs.

A recent genotypic study [62] showed that development of INSTI-resistance mutations is not restricted by drug-resistance mutations in the PR and/or RT coding regions; however, Buzon *et al* [48] described that although mutations outside the integrase coding region may not affect susceptibility to INSTIs, mutations within the PR and RT rescued the replication capacity of viruses with INSTI resistance mutations, suggesting the existence of epistatic effects on HIV-1 replicative fitness. Epistasis in RNA viruses, particularly positive gene interactions in HIV-1, has been associated with mutations affecting fitness [63], [64]. Here we compared the susceptibility of two sets of recombinant viruses, constructed from patient-derived *gag*-p2/NCp7/p1/p6/*pol*-PR/RT/IN or IN fragments, to RAL and EVG and demonstrated that mutations in the protease and RT have limited effect on susceptibility to these INSTIs. The p2-INT-recombinant viruses engineered from 27 highly treatment-experienced patients carried a multitude of drug resistance mutations, in different combinations, which generated diverse levels of drug susceptibility and yet none of them significantly affected the sensitivity of the virus to RAL and EVG. On the other hand, the replicative fitness of the p2-INT- recombinant viruses was different, and in most cases reduced, when compared to that of the INT-recombinant viruses. Epistasis is positive when it enhances the fitness predicted from individual effects of deleterious (e.g., drug resistance) mutations and is negative when it decreases fitness [65]. Unlike the positive epistasis previously reported in HIV-1 [48], [63], [66], i.e., mutations in other genomic regions restore viral fitness impaired by mutations in a given gene, we show that drug resistance mutations in the PR and RT contribute to decrease the replicative fitness of the virus already impaired by mutations in the integrase. This negative epistasis indicates that HIV-1 is highly sensitive to the combinatory effects of deleterious mutations in different genes, as described for HIV-1 protease [67] and gp120 [66]. However, it is important to note that in this study the negative epistasis associated with a reduction in viral fitness refers to an environment characterized by the absence of drug pressure. The otherwise deleterious (drug resistance) mutations confer a distinctive advantage in the presence of antiretroviral drugs, increasing the replicative fitness of the virus in this new environment and consequently changing the “sign” of the epistasis to positive [63]. Thus, epistatic interactions and their role in HIV-1 replicative fitness is complicated and depends on environmental factors such as the absence or presence of drugs [68].

In summary, our novel HIV-1 phenotyping assay based on patient-derived *gag*-p2/NCp7/p1/p6/*pol*-PR/RT/IN fragments was not only able to simultaneously quantify susceptibility to protease, RT, and integrase inhibitors but to also measure the effect of multiple drug resistance mutations on viral replicative fitness. Mutations in the PR and RT-coding regions do not seem to have a significant effect on susceptibility to INSTIs; however, all HIV-1 genes are involved in modulating viral replicative fitness, particularly those regions carrying drug resistance mutations. Therefore, the use of recombinant viruses containing most or all HIV-1 regions targeted by antiretroviral drugs seems to resemble better the characteristics of the actual virus circulating *in vivo* and might be key in future studies aimed at understanding the potential collective effect of epistatic interactions in multidrug resistant viruses.

## Supporting Information

Table S1 **HIV-1 genotype for the 27 HIV-infected individuals participating in the GS-US-183-0105 study of elvitegravir.**
(DOCX)
