# Supplementary material for: Resistance Mutations outside the Integrase Coding Region Have an Effect on Human Immunodeficiency Virus Replicative Fitness but Do Not Affect Its Susceptibility to Integrase Strand Transfer Inhibitors
Source: PLoS One. 2013 Jun 11;8(6):e65631. doi: 10.1371/journal.pone.0065631 (PMC3679210; doi:10.1371/journal.pone.0065631)
Supplement: Table S1 — HIV-1 genotype for the 27 HIV-infected individuals participating in the GS-US-183-0105 study of elvitegravir. (DOCX) [file pone.0065631.s001.docx]

**Supplemental Material Table S1.**  HIV-1 genotype for the 27 HIV-infected individuals participating in the GS-US-183-0105 study of elvitegravir.

| **Group** | **Patient** | **Protease Sequence** | **RT Sequence** | **Integrase Sequence** |
| --- | --- | --- | --- | --- |
| E92Q | 08-186 | L10I D30N L33I E35D M36I N37S R41K R57K I62V L63Q I64V A71V V77I G86G/R N88D | K32K/T V35K T39A M41L V60I D67G S68G K70R K101E Q102K K103N K122E D123E E138Q C162S D177E I178L V179I/T Y181C M184M/I D192D/N G196E Q207N R211Q T215F K219E L228H Q242H V245E S251N R277K L283I K287R V292I | V31I E92Q L101I V113I T122T/I I151V K156N V234L S255R |
|  | 08-196 | L10I I13V M36I N37S R41K D60E Q61E L63P I93L | P4S T7P V35L T39A M41L K43Q E44D D67N T69D Q102K K103N V106V/I V108I D123E C162S D177E M184V T200E L210S T215Y F227L M230L A272P T286A | D3D/E E11D K14R R20K V31I C56C/R K71K/R V72I E92Q L101I V113I S119P I135V I151V G193E V201I T218S I220V V234L D256E V260V/A |
| E92Q + L68V/I | 08-180 | L10I I13V I15V K20T V32I L33F E35E/K/EE/KE M36L R41K K43T M46I I47V I54L I62V L63P I66F A71V G73S V82V/A/I/T I84V L89V L90M | K32K/E V35I M41L E44D S48T D67N V75M F77L A98S Q102K V118I K122P I135T C162S D177E M184V H208Y L210F T215Y K219N V261V/I A272A/P R277K A288A/T E297K | E11D K14R V31I L68L/V V72I E92Q L101I T112T/I V113L N117N/K I151V V201I T206T/S I208L V234L K240K/R D256E |
|  | 08-195 | L10I V32I L33F E34Q E35D M36L N37E R41K I47V G48V I54M L63P A71V V77I V82A L90M I93L | M41L D67N K70Q L74V A98A/G K101E Q102K V108V/I V111I V118I K122E I142V C162S R172K Q174E/K Y181C M184V G190A E203D Q207K H208Y L210W R211D T215Y K219R H221Y L228H K238T A272P R277K T286A E297R | E11D D25D/E V31I L68L/V V72I E92Q V113I I151V M154L V201I V234L |
|  | 08-223 | L10I V11L I13V Q18H L19I L33F N37D K43T M46I/L F53L I54L L63P A71V I72L G73S T74A V77I T80I L89V L90M I93L G94R | E6K K11K/E K43K/E T58T/A A62V D67E S68T T69SSS L74V Q102K K122E A158S C162S V179I M184V T215F L228H L234L/F V245E R277K Q278H E297Q L301L/P | R20K V37V/I E48E/G M50M/V H51H/Y L68L/I/V V72I E92Q L101I K111T T124T/N V126V/A I151V V176L Y194Y/H E198E/K V201I T206S I220I/V V234L D253D/N D256E |
| E92Q + N155H | 08-202 | I15V P39P/L R41R/K M46M/I I50I/V I54I/L Q61E L63P A71V V77I L90M I93L | K20R M41L R83R/K Q102K K103N K122P I135T C162S D177E I178M G196E R211G T215Y K249K/E A272P R277K E297A | K14R L28I S39C E92E/Q L101I K103K/R V113I T124A I151V N155N/H I200M V201I V234L V249V/I D253E N254G |
|  | 08-230 | L10I I13V K20K/R L24I L33F M36M/I R41K M46M/I G48M I54S I62V L63P A71L I72V V77I V82A I84V I93L | E6E/K K20R M41L V60I D67N T69N K70R L74I K101S Q102K K103N K122E N136N/D C162S E169E/G D177E I178L M184V D192D/N G196E Q207H R211K T215F D218E K219Q A272P R277K P294Q E297S | S17N V72I E92Q L101I V113I F121F/S T124T/S I141I/V I151V N155H F181L K188R T206S I220L V234L N254N/S I268L R269K/R D270H |
| E92Q + L68V + N155H | 08-210 | L10I/V V11I I13V V32I L33F E34Q N37D R41R/G K43T M46L I54L K55R D60D/E L63P A71V V82A I84V L90M Q92R | V8I K20R M41L E53D D67N K70R L74I V75T A98A/S Q102K V108I K122P Q151Q/R C162S I178M V179V/I Y181C M184V G190A T200A E203D E204E/K Q207E L210W R211R/K T215F K219W L228H A272P T286A V293I E297R | E13D S17N V31V/I L68L/V V72I E92E/Q L101I K103K/R V113I T125I/M/V A128T I151V N155N/H K156N V234L Q252K D288N |
| E92Q + T66I/A | 08-198 | I13V I15V L19T K20T E35E/D L63P | K20R P25P/L K32K/R K49R I50I/V Q102K V118I I135T C162S T165I M184V T200A H208Y R211K F214L T215F A272P R277K R284K V293I E297K | S17S/N T66T/A L68L/I V72V/I E92E/Q L101I K111T V113I E138D S147S/G I151V K159K/E T206S E212A D232D/N V234L D253E Q274Q/R S283G |
|  | 08-199 | L10I I13M K20V L23I E35G M36I M46I I50V F53F/L R57K I62V L63P I64V I72V G73I V82I I85V L90M T91T/I | P4S K20R T39A M41L G45G/E D67N K70R Q102K K122E D123E/G C162Y M184V H208Y R211K T215F K219Q E224D H235H/R V245K A272P R277K A288T V293I E297K | G4G/E S17N R20K T66T/I V72I/T E92E/Q L101L/I K111T V113I S119P T124G T125A I151V T206S V234L D279D/A/N/T S283G |
| N155H | 08-245 | E35D N37S L63T I64V I72V V77I V82I | Q102K K122E I142I/V C162S M184V R211K F214L A272P R277K T286S | E11D S24N V72I V113I I151V M154L N155H E170A K211R T218I V234I S283G |
|  | 08-240 | L10F I13V V32I M36L N37N/D L38W P39Q M46I I47V I50V I62V L63P A71I I72V V82A | M41L K43E E44E/A/D K64R D67N Q102K V111V/I/M V118V/I K122E D123D/N C162S E169E/D M184V E194D G196E I202I/V H208F L210W R211R/K T215Y A272P R277R/K R284K T286A E297K | V72I L101I V113I A128A/T I135I/V I151V N155H K156R D167D/N K188R T206S I208L K211K/E D232D/H V234L A265A/V I268I/L R269R/K D270D/N |
| N155H + S119P/R | 08-194 | L10I I13V L33F E35N N37E R41K M46I K55R R57K I62V L63P T74P V77V/I V82V/I I84V I85V L89M | K20R M41L S68S/G L74I V75T W88W/C A98G Q102K K103K/N V118I K122E C162S M184V R211K F214L T215F V245K V276I R277K L283I A288S V293I E297R A304E | E10D V72I P90A A91S L101I V113I S119R T124A E138E/K I151V N155H K156N V201I K211R V234L |
|  | 08-201 | I13V K14R V32I N37S R41K M46I I47V L63P I72V G86G/E | K11K/N/R/S V35V/I/M Q102K V118V/G D121Y K122E I135T K154K/R M164M/T P170P/T M184V L210F R211K A272P R277K P294A/S E297K A304E | K7Q E11D N27G V32I D41N V77V/A A91R L101V T112A V113I S119P T124N T125A I151V M154L N155H R166R/S T218S V234I |
| N155H + S230R | 08-174 | L10I T12P I15V Q18F L19E K20T E35D M36I I50V I54A L63P A71V G73S V77I I85V L89V L90M | M41L E44D K49R V60I D67N T69D V75M A98S L100I Q102K K103N V118I K122E I135T C162D K166R Q174K M184V G196E T200E E203K Q207E L210W R211K T215Y K219N V245M A272P R277K E297R | E11D A21T A23V M50I L101I T112I/V V113V/L S119P T122T/I T124A I151V N155H K156K/R D167E V201I T218I K219N N222K S230S/R V234I D288N |
| Q148R | 08-184 | L10I I15V E35D M36I N37D R57K L63P T74A I93L | D67N V90I Q102K K103N V108I D123E I135M C162A I178M M184M/I/V Q207E R211K F214F/L T215F K219R P225H A272P R277K | V72I Q148R I151V S230N D232E V234L D256E R284G |
| Q148R/E138K/S147G | 08-182 | L10I I15V L33F I54M Q61H V82I I84V L89F | K65R S68G L74I R83K K101P Q102K K103S D123E C162S K173D/N Q174K I178L M184V D192D/N R211K V245E D250E S251D A272P V293I | G106A K111T V113I T124N E138K S147G Q148R I151V Y194C K211R N222K S230N V234L |
|  | 08-209 | T4S L10F V11L I15V K20V V32I L33F E35D M36I N37D K43T M46I I54L I62I/V L63L/P A71I I72K G73T T74T/P I84V I85V L89V L90M | V21I V35L S48T T58N A62V D67G S68G K70E V75I F77L K101E Q102K V106I Y115F F116Y K122E D123D/G/N/S Q151M C162N S163S/G Q174K Y181C M184V G190S G196K Q197Q/R T200E R211K F214L K223K/R V245M A272P R277K P294P/L | V72I V79V/I V113I E138K S147G Q148R I151V K160K/N S195C I203M V234L R284G D288G |
| Q148R/H/K + G140S/C | 08-197 | L10F I13V L24F L33F R41K M46I D60E I62V L63P A71V G73S/T P79A I84V L90M I93L | K20R E36E/K T39A M41L V60V/I D67G S68G K70R L74L/I Q102K K103N D121Y K122E C162S Q174R D177E M184V T200A Q207E F214F/L T215F K219Q P225P/H L228H V245I A288S | D6D/N S17N E35Q L101I V113I T124N K136Q G140G/C Q148R I151V F181L V201I V234L A265V D288N |
|  | 08-239 | L10I T12P K20R V32I L33F E35D M36I R41H K43T M46L K55R I62V L63T I64L A71V I72T T74S V77I V82A I84V L89M L90M I93L | E6E/K K20K/R V21V/I V35M T39T/A M41L K43N E44D K49K/R V60I D67N T69D V75M Q102K K103N V118I K122E I135L C162D K166R D177E I178M M184I T200A E203K Q207E L210W R211K T215Y K219R K223E Q278E T286T/A A288A/S V293I E297K L301L/F | K14K/R S17N V31I V72I V113I T124A G140S Q148H I151V K188R V201I I220L V234L D253E |
| Q148R/H/K + N155H | 08-205 | L10I I15I/V K20R A22V L24I M36I N37D K43T M46I G48M I54V L63P A71V V82T I84V T91A/S | V8I V35E T39A M41L E53D D67N T69N K70R A98G K101K/R Q102K V108V/I K122E D123E C162H Q174R V179I M184M/V T215F K219E L228H A272P R277K T286A A288T V292I E297R | V72I L101I G106A V113I S147S/G Q148Q/R I151V N155N/H G193E V201I T206S E212G S230S/R D232D/N V234L D256E D288D/N |
|  | 08-232 | L10I I13V L24I V32I L33F M36L R41K K43T M46L I47A I62V L63Q I64V E65D I72I/V V82A | M41L K43N D67N A98S Q102K K103N V118I K122E D123S I135T C162S K166R V179I M184V V189V/I T200R I202V L210W T215Y K219K/R V245K A272P T286T/A E297Q | S24S/G S39C L45Q Y99Y/F K111K/R V113I T125V E138E/K Q148Q/R I151V N155N/H R166R/S E170E/A V234L K240K/R |
| T66I/A + other mutations | 08-175 | L10F/I K14R L33L/F E34Q E35E/D M36M/I/V M46M/I I54I/L/V Q58Q/E L63P I64V I66I/V I72I/M G73T I84V L90M I93L C95F/V | P9P/S V35M T39A E40F/S M41L K43E D67N L74V L100I Q102K K103N V118V/I K122E M184V T200T/A H208Y L210W R211K T215Y D218E K219N L228H V245E E248D A272P T286T/A A288S V293V/I E297K | S17N V31I T66T/A/I/V I73V L101I V113I T124N S147S/G I151V V234L A265A/V |
|  | 08-193 | L10R T12P I13V L19Q L23I L33F M46L K55R Q58E L63P I64V V77I V82T I84V L89L/M | M41L V60I D67N T69D K70R A98G Q102K I135T C162S K173K/R I178M M184V G196E R211T T215F K219Q L228H V245Q S251H R277K T286A V293I E297A | S17S/N V31V/I V32I D41G H51H/Y T66T/A V72I L101I K111T T112T/A T124N T125A E138E/K Q146Q/L S147G I151V F181L V234L |
| Other INSTI-R  (no 66, 92, 148, 155) | 08-200 | L10I I13V I15V K20R L33F E35D M36I N37E M46I I54V K55R R57K Q58E I62V L63P A71L L76I V82V/L L89M L90M I93L | P1L A62V S68G K70N V75V/M F77L V90I Q102K K103N V108I F116Y K122E I135L Q151M C162D M184V T200A E203D Q207A R211K T215Y K223T L228R R277K L283I R284K V292I | K7Q E11D D25E V31I V72I L101I V113I S119P F121C T122I T124A I151V D232N V234L |
|  | 08-236 | L10F L33F E34E/Q R41K M46I I47V I54M K55R I62V L63P C67F A71V I72L G73T I84V I85V L90M | K20R E29E/G K30R V35L T39A M41L D67N T69D K70R L74I V75T Q102K K103N K122E D123S C162D V179I Y181C M184V G196E T200A E203D T215F D218E K219Q L228H K238R Q242H V245E A288S E297A | V31I P90S K111T V113I T124N T125V V126M E138E/K Q146I I151V Q216Q/R D232D/N V234L D256E |
| No INSTI-R mutations | 08-179 | L10I I13V L33F M36M/L N37S R41K/N M46L G48G/E/V I54I/V Q58Q/E I62V L63P I64V T74P V82A I84V | K20K/R V35V/I/M M41L K43N E44D D67N W88W/R A98G Q102K V108V/I V118I I135L C162S M184I T200T/A E203K H208Y L210W R211K T215Y K219R K223E F227L A272P K281K/R T286A V293I E297K | E10A E11D S17N V31V/I M50T T112V V113V/I T125A V126V/M I151V K156N V201I V234L D256E |

Patient-derived protease, RT, and integrase PCR products were originally sequenced using eight overlapping sequencing primers in an ABI37370 automated sequencer and compared to the reference HIV-1_NL4-3_ ([1](#_ENREF_1)). PCR products corresponding to the *gag*-p2/NCp7/p1/p6/*pol*-PR/RT/IN-coding region from the same patients were sequenced to confirm the original sequences using AP Biotech DYEnamic ET Terminator cycle with Thermosequenase II (Davis Sequencing LCC, Davis, CA). Nucleotide sequences were analyzed using DNASTAR Lasergene Software Suite v.7.1.0 (Madison, WI).

**REFERENCES**

1. **McColl DJ, Fransen S, Gupta S, Parkin N, Margot N, Chuck S, Cheng AK, Miller MD.** 2007. Resistance and cross resistance to fist generation integrase inhibitors: insights from a phase II study of elvitegravir (GS-9137). Antiviral Therapy **12:**S11.
